# Unmasking the rarity of mammary analogue secretory carcinoma of the minor salivary gland - A case report

**DOI:** 10.1016/j.ijscr.2024.110374

**Published:** 2024-09-30

**Authors:** Samkit V. Sakhariya, Anuja Chincholkar, Sanika Tidke, Sneha Setiya, Noopur Mane, Megha Markand

**Affiliations:** a Dept. of Oral and Maxillofacial Surgery, Dr. D. Y. Patil Dental College and Hospital, Dr. D.Y. Patil Vidyapeeth, Pune, Maharashtra, India; b Indrayani Hospital & Cancer Institute, Pune, Maharashtra, India; c Dept. of Oral and Maxillofacial Surgery, M. A. Rangoonwala College of Dental Sciences & Research Centre, Pune, Maharashtra, India; d Pune, Maharashtra, India; e Dept. of orthodontics and dentofacial orthopaedics, M. A. Rangoonwala College of Dental Sciences & Research Centre, Pune, Maharashtra, India

**Keywords:** Mammary analogue secretory carcinoma, Minor salivary gland, Potentially low-grade malignancy, Immunohistochemistry, Secretory carcinoma breast, Neck dissection

## Abstract

**Introduction:**

Mammary analogue secretory carcinoma (MASC) of the salivary gland was first described by Skálová et al. in 2010. It is often associated with a translocation, t(12;15)(p13;q25), which results in the fusion gene ETV6-NTRK3. Major salivary glands, primarily the parotid gland, are involved in 70 % of cases of MASC, while small salivary glands are involved in less than 25 % of cases. This report aims to consolidate in unveiling, diagnosing, and managing the rarity of MASC in the minor salivary gland and its existing knowledge and encourage new research on this increasingly important salivary gland malignancy.

**Presentation of the case:**

A 27-year-old female reported with a complaint of swelling on the right cheek region of face since 10 weeks. On bimanual palpation, a soft lobulated mass was appreciated beneath the healthy mucosal layer. The radiographic image (orthopantomogram) showed no obvious calcified mass. An excisional biopsy was planned and performed under local anesthesia. Microscopic and immunohistochemistry confirmed the tumor to be a MASC of minor salivary gland.

**Discussion:**

Due to their infrequency and multiplicity of histopathology, MASC presents difficulty in diagnosis. A key to determining diagnostic criteria for MASC is to study cellular morphology, cytoplasmic filament expression, and ultrastructural features of the tumor and apply this information to defining MASC.

**Conclusion:**

MASC is an important molecularly defined entity of the salivary gland with low-grade malignant potential. Correct diagnosis is essential for appropriate treatment and will help to provide better information about this potentially low-grade malignant salivary gland neoplasm.

## Highlights

• Mammary analogue secretory carcinoma of minor salivary gland
• Electron microscopy and Immunohistochemistry
• Low-grade carcinoma of salivary glands
• Secretory carcinoma of breast

## 1 Introduction

Mammary Analogue Secretory Carcinoma of salivary glands (MASC) is a low-grade carcinoma of salivary glands of the head and neck region and was first described by Skálová et al. in 2010. It was reported in the 4th edition of the WHO classification of head and neck tumors. Major salivary glands, primarily the parotid gland, are involved in 70 % of cases of MASC, while minor salivary glands are involved in less than 25 % of cases [1]. Clinically, MASC presents as a slow-growing mass with no pain and seldom any pus discharge. In cases involving the parotid gland, pain, and facial paralysis have been reported. MASCs are in general indolent, solitary, well-circumscribed masses that are primarily unencapsulated and have a cut surface that is white, grey, brown, or yellow. In certain cases, a substantial cystic component has been noted in these tumors. It bears histological resemblance to the Secretory Carcinoma of the breast and Acinic Cell Carcinoma of the parotid gland [2]. Despite distinctive histologic features, MASC may be difficult to distinguish from other salivary gland tumors, particularly acinic cell carcinoma and intraductal carcinoma [3]. The repeated balanced chromosomal translocation t(12;15)(p13;q25), which results from the fusion of the ETS Variant Transcription Factor 6 (ETV6) gene on chromosome 12 and the Neurotrophic Receptor Tyrosine Kinase 3 (NTRK3) gene on chromosome 15, has been associated with both MASC and secretory breast carcinomas [4]. Using death or recurrence as the endpoint, the mean disease-free survival is reported to be 92 months [5].

This report aims to consolidate in unveiling, diagnosing, and managing the rarity of MASC in the minor salivary gland and its existing knowledge and encourage new research on this increasingly important salivary gland malignancy.

## 2 Case report

**Fig. 1:**
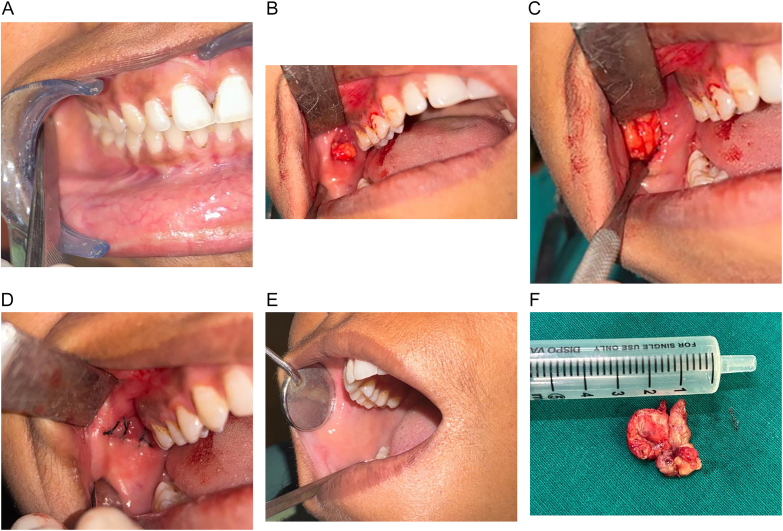
Clinical pictures. A. Presentation of healthy mucosa. B. Initial intra-operative picture of the lesion. C. Intra-operative picture of the lesion. D. Primary closure. E. 6-months follow-up picture. F. Excisional specimen.

A 27-year-old female reported to the Department of Oral and Maxillofacial Surgery of our institute, complaining of swelling and pain in the right cheek region of face since 10 weeks. The patient gave no history of any known medical condition or co-morbidities. The tenderness increased at the time of mastication. The physical local examination revealed no signs of inflammation, fibrosis, ulceration, discharge, or color change (Fig. 1A). Although on bimanual palpation, a soft lobulated tender mass of 1.5*1 cm size was appreciated beneath the mucosal layer in the right buccal mucosa region. The patient was advised to undergo a radiographic investigation (orthopantomogram) to rule out any bony pathology. The radiograph showed no radio opacity. This case report has been reported in line with the SCARE criteria (2023) [6].

**Fig. 2:**
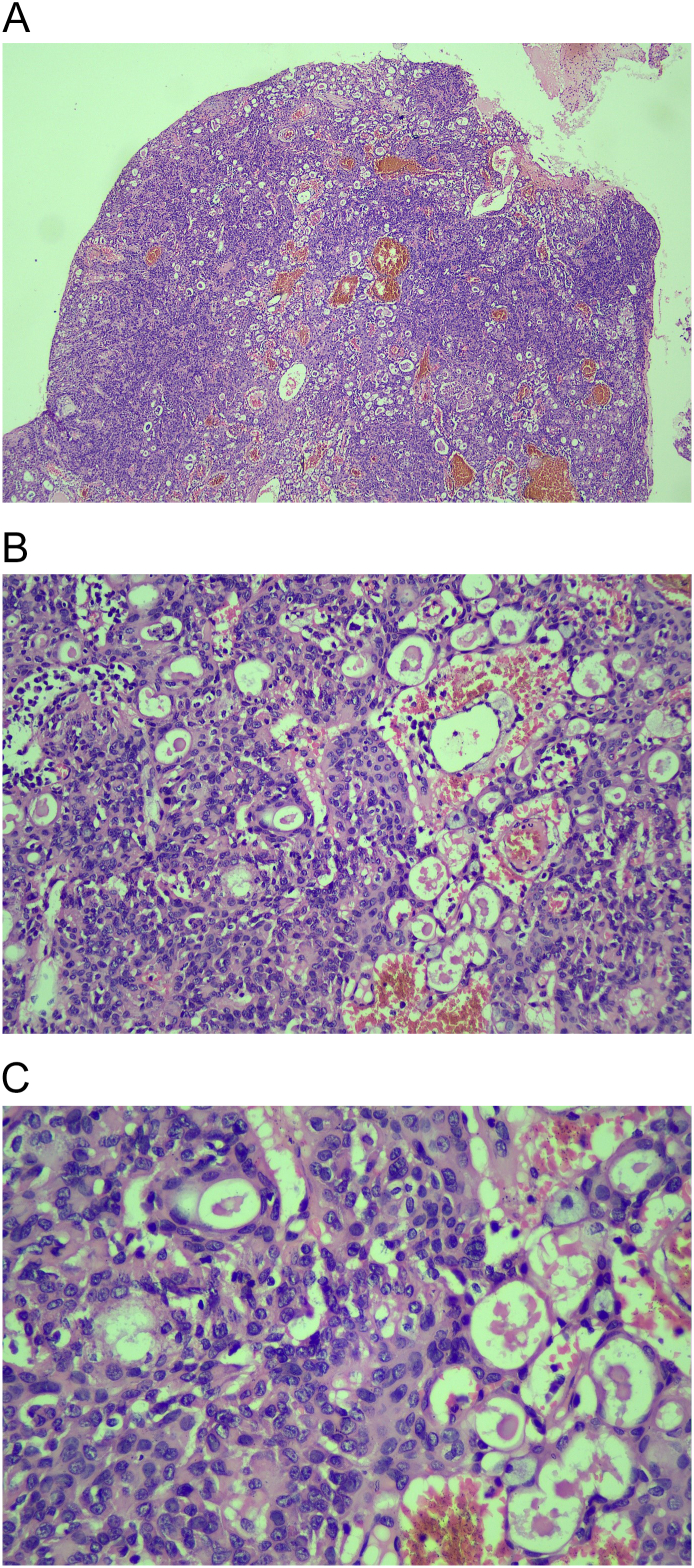
A-C. Histopathology Sections – A solid nodular tumor with lobulated, follicular and focal invasive areas. Neoplastic cells are monomorphic epithelioid. The follicles contain colloid-like eosinophilic secretions.

**Fig. 3:**
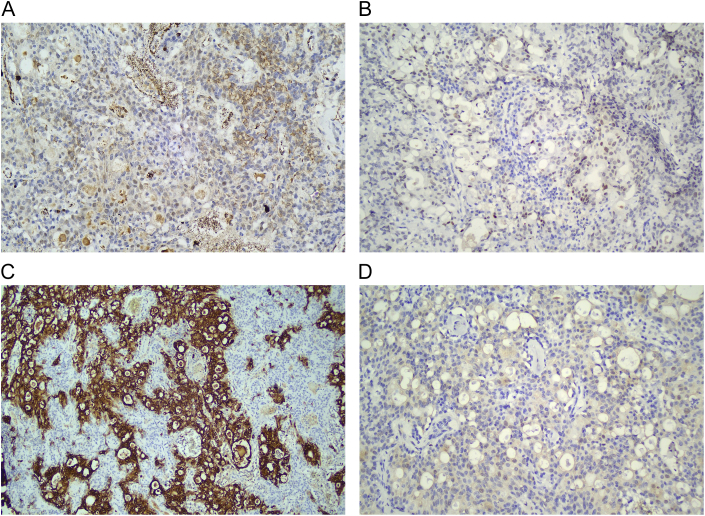
Immunohistochemistry slides. A. CK-7 positivity in the luminal cells. B. Diffuse staining for MUC-4 in the tumor cells. C. Nuclear positivity for GATA-3 in tumor cells. D. SOX-10 positivity in tumor cells.

The patient was scheduled for an excisional biopsy under local anesthesia with all the valid informed written consents obtained. A complete pre-operative surgical blood profile report was obtained for fitness. After infiltrating 2 % lignocaine with 1:200,000 adrenaline, Stenson's duct orifice was identified and marked. A horizontal incision was made on the buccal mucosa 5 mm below the orifice, parallel to the occlusal plane. Blunt dissection was done and a well-circumscribed lesion was identified and excised. Hemostasis was achieved and mucosal closure was done in a single layer (Fig. 1B-D). On gross examination, the surface of the lesion appeared to be yellowish with well-defined borders. The lesion was rubbery in consistency with a solid and lobulated appearance, measuring about 2.2*1.5 cm in size (Fig. 1F), and later subjected to microscopic and immunohistochemistry examination. Microscopic examination revealed the presence of nodular tumor with lobulated, follicular, and focal invasive areas. Neoplastic cells were monomeric epithelioid with vacuolated eosinophilic cytoplasm and a central round nucleus with a small nucleolus. The follicles contained colloid-like eosinophilic secretion and focally mucoid contents. There was no evidence of mitoses or necrosis (Fig. 2A-C). The Immunohistochemistry (IHC) report suggested that neoplastic cells were positive for SRY-related HMG box 10 (SOX-10), **GATA binding protein 3** (GATA-3), and **Mucin 4** (MUC-4) stains, they were negative for Cytokeratin 7 (CK-7) (Fig. 3A-D). The final impression was suggestive of MACS of the minor salivary gland.

**Fig. 4:**
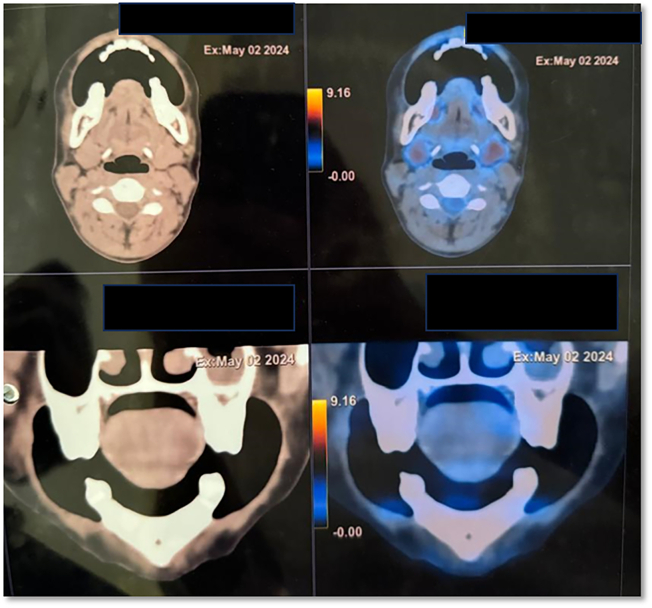
PET-CT - no FDG avid lesion noted in right buccal mucosa.

**Fig. 5:**
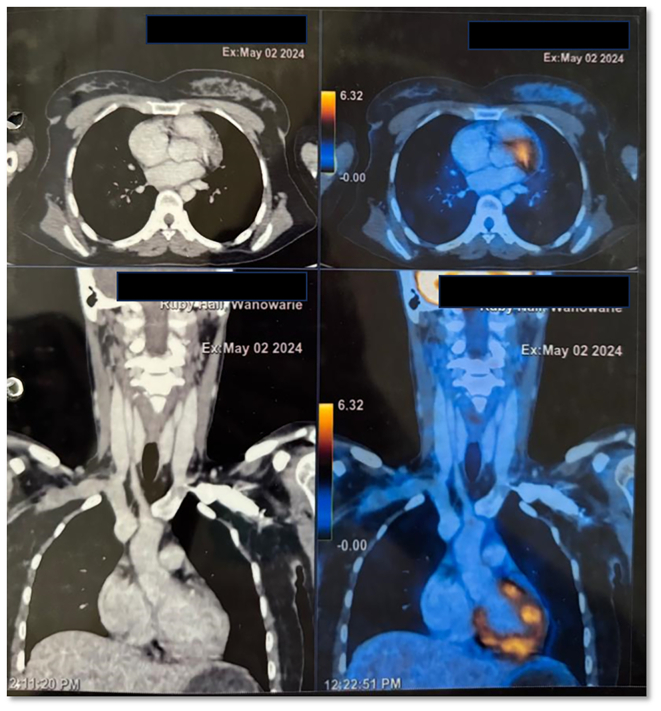
FDG uptake (SUVmax 3.2) noted in upper inner quadrant of left breast.

Since it is a neoplastic lesion, the patient was advised for Positron emission tomography-computed tomography (PET/CT) after 3 months, which suggested no fluorodeoxyglucose (FDG) avid lesion in buccal mucosa (Fig. 4), however, a low-grade FDG uptake (SUVmax 3.2) was noted in upper inner quadrant of left breast was noted with no enlargement of local lymph nodes (Fig. 5). Patient was the scheduled for right Supra-omohyoid neck dissection (SOHND) and excision of left breast tissue excision under general anesthesia. On histopathologic examination, the nodes showed no signs of malignancy and excised breast tissue was reported to be benign. No clinical signs of recurrence are observed at the 6-month follow-up (Fig. 1E), and the patient is advised to continue monthly follow-up.

## 3 Discussion

Mammary analogue secretory carcinoma (MASC) is a rare salivary gland tumor that has a similar histological and genetic profile as that of a rare malignancy of the breast–secretory carcinoma. The tumor may appear as a sizable cystic mass with a multilayered lining that may have papillary, macro-cystic, or microcystic architecture, as well as tubular, follicular, and occasionally solid regions [7].

MASC often affects patients in their fourth to fifth decade of life. Clinically, the patient presents with a slow-growing mass, which is most often non-painful. In our case, the patient only reported swelling and occasional pain on mastication, with no signs of any facial nerve palsy. Facial nerve palsy is at times noted in patients with MASC affecting the parotid gland. Histologically, misinterpreting MASC as pleomorphic adenoma, mucoepidermoid carcinomas, adenocarcinoma not otherwise specified (NOS), intraductal carcinomas, or acinic cell carcinomas (ACC) may lead to false and inadequate therapeutic intervention [8].

The histopathologic pattern of these tumors is distinctive as they are well-circumscribed, at least partially encapsulated. The tumor nodules are made up of epithelial nests that are arranged in tubular and tubulo-cystic, papillary, and cribriform patterns, occasionally containing foamy histiocytes mixed in with them, and an abundance of intraluminal secretory material (Alcian-PAS stain positive). Tumor cells also exhibit moderate cellular pleomorphism, oval to round nuclei, eosinophilic cytoplasm, and moderate cell size [8,9]. The histopathology examination of our specimen showed the presence of a solid nodular tumor with lobulated, follicular, and focal invasive areas. The cytoplasm was vacuolated eosinophilic in nature with a central round nucleus. The nucleolus was small but distinctive.

Khurram et al. suggested that the immunohistochemistry technique can better and can specifically detect MASC and differentiate it from acinic cell carcinomas or pleomorphic adenoma [10]. Up to a 10 % proliferation rate has been determined in the existing literature. Positive results are seen with SOX-10, GATA-3, vimentin, and mammaglobin stains, whereas, consistently negative results for hormone receptors (progesterone and estrogen), Her2/neu, p53, prostate-specific antigen (PSA), and androgen receptor (AR) have been reported in various studies [[8], [9], [10]]. In the present case, immunohistochemistry showed positivity to SOX-10, GATA-3, and MUC-4 but showed negativity to CK-7.

Although characteristic morphologic and immunohistochemical features form the basis of a diagnosis of MASC, Fluorescence In-Situ Hybridization (FISH) screening for the rearrangement of ETV6 gene locus and the presence of an ETV6-NTRK3 gene fusion is considered as confirmatory [3,11,12]. The repeated balanced chromosomal translocation t(12;15)(p13;q25), which results from the fusion of the ETS Variant Transcription Factor 6 (ETV6) gene on chromosome 12 and the Neurotrophic Receptor Tyrosine Kinase 3 (NTRK3) gene on chromosome 15, has been associated with both MASC and secretory breast carcinomas [4].

MASC most often resembles acinic cell carcinoma, which contains large serous acinar cells with cytoplasmic PAS-positive zymogen-like granules. The major differentiating feature of MASC is the absence of acinar cells and zymogen-like granules [11]. Another differentiating histopathologic entity is the intraductal carcinoma, which shows an intact myoepithelial rim around tumor nests, which is not seen in MASC [11].

Since these tumors are neoplastic, a more aggressive therapeutic approach is often suggested once the diagnosis of MASC is confirmed by histopathologic, IHC, and molecular analysis.

Literature shows that patients with MASC undergo varying degrees of surgical treatment. These include surgical resection, neck dissection (depending on the reported/associated cervical lymphadenopathy), postoperative radiotherapy, and a combination of radiotherapy with chemotherapy [13].

MASC should be treated as nothing less than any aggressive malignant salivary gland tumor with a proper follow-up to assess for recurrence and quality of life post-treatment.

As this is a rare new entity, not routinely encountered, and there are no established pre-operative diagnostic guidelines, it can be confused with another buccal mucosa swelling, myoepithelioma, or salivary sialolith as happened in this report. With this case report, we intend to spread awareness of this rare entity and its consideration for differential diagnosis, highlighting the importance of pre-operative definitive investigations like Ultrasonography-USG, Fine needle Aspiration Cytology-FNAC, and Contrast Enhanced Computed Tomography-CECT for accurate diagnosis. This will also provide ease in formulating the treatment plan and reduce repetitive hospital exposure.

## 4 Conclusion

MASC is a newly recognized malignant salivary gland tumor distinct from acinic cell carcinoma that many times mimics the histology and genetics of secretory carcinoma of the breast. Although it appears to follow an indolent course in most patients, certain cases appear predisposed to distant metastasis and increased mortality. MASC shows the usual histological features of the nodular tumor with lobulated, follicular, and focal invasive areas, high immunohistochemistry staining for S-100 and mammaglobin, and a distinctive ETV6–NTRK3 gene fusion. There is a growing body of data on this disease in the pathology literature. Further research is needed to better delineate its overall prevalence, understand the clinical behavior and prognostic significance of MASC, to define an appropriate diagnostic and treatment algorithm. Indeed, large-scale retrospective analysis of existing salivary gland carcinoma tissue may help to answer this question.

## Consent

Written informed consent was obtained from the patient for publication of this case report and accompanying images. A copy of the written consent is available for review by the Editor-in-Chief of this journal on request.

## Ethical approval

The case was performed at the same institution hence, ethics approval is not required for case reports or case series deemed not to constitute research at the institution hence it was waived by the authors' institution(Dr. D. Y. Dental College and Hospital) Name of ethics committee -Dr. D. Y. Patil Vidyapeeth, Pune. (Reg. No. ECR/361/Inst/MH/2013).

## Funding

None.

## Author contribution

Dr. Samkit Sakhariya – Study concept or design, data collection, manuscript writing, editing and reviewing.

Dr. Anuja Chincholkar - data collection, manuscript writing, editing, and reviewing.

Dr. Sneha Setiya – manuscript reviewing.

Dr. Sanika Tidke – data analysis, manuscript editing and reviewing.

Dr. Noopur Mane - Supervision, validation.

Dr. Megha Markand - Supervision, validation.

## Guarantor

Dr. Samkit Sakhariya is the guarantor and takes responsibility of the data originality and conduct of study.

## Research registration number

N/A.

## Conflict of interest statement

None.
